# TNF‐α–induced p53 activation induces apoptosis in neurological injury

**DOI:** 10.1111/jcmm.15333

**Published:** 2020-04-28

**Authors:** Xuefei Shao, Xiping Yang, Jun Shen, Sansong Chen, Xiaochun Jiang, Qifu Wang, Qiang Di

**Affiliations:** ^1^ Department of Neurosurgery Yi‐Ji Shan Hospital of Wannan Medical College Wuhu China; ^2^ Characteristic Medical Center of the Chinese People's Armed Police Force Tianjin China; ^3^ Department of Dermatology and STD Yi‐Ji Shan Hospital of Wannan Medical College Wuhu China

**Keywords:** apoptosis, apoptotic signalling pathway, p53, TNF‐α, traumatic brain injury

## Abstract

It was previously confirmed that the apoptotic and necrotic neurons are found during the acute post‐traumatic period, suggesting the induction of apoptosis after traumatic brain injury (TBI). To further explore the involvement of apoptotic factors in TBI, an apoptosis antibody array was conducted to measure the alterations of apoptotic factors in rat brain cortex after TBI. As a result, the Neurological Severity Scale (NSS) scores after TBI were increased, and the cell morphology of the brain cortex was destructed with increased neuronal apoptosis. Furthermore, the caspase‐3 activity was increased, and the apoptotic‐related factors TNF‐α and p53 were up‐regulated in the brain cortex. More importantly, in vitro experiments demonstrated that down‐regulation of TNF‐α in oxygen‐glucose deprivation/reoxygenation (OGD/R) cells increased cell viability and decreased apoptosis and the p53 expression. These results suggested the involvement of TNF‐α–induced apoptotic signalling pathway by activating p53 in the molecular mechanism of neurological injury.

## 1 INTRODUCTION

Traumatic brain injury (TBI) is an injury to the brain caused by an external force, leading to the destruction of brain functions, such as motor movement, learning and memory neurobehavioural deficits. TBI survivors often have physical, emotional and behavioural problems, which subsequently leads to a burden on the healthcare system and effect on the life quality. Although there are many approved drugs for the clinical therapy of TBI, most of these appeared ineffective. Therefore, it is necessary to understand the molecular alteration after TBI in order to provide specific targeted therapeutic strategies. In normal brain, the physiological homeostasis is maintained by endothelial cells, neurons and glial cells.^1^, ^2^ However, in TBI survivors, a series of pathophysiological processes including neuroinflammation and apoptotic cell death can be triggered. TBI causes cell shearing and membrane rupture, irreversible cell injury and necrosis.^3^ Therefore, apoptotic and necrotic neurons are observed in the acute post‐traumatic period, including the apoptotic characteristics of cell shrinkage, cytoplasmic blebs and DNA fragmentation. Apoptosis is the process of morphological manifestation of programmed cell death and is initiated by either extrinsic or intrinsic signals, which generally requires synthesis of new RNAs and proteins to suppress or promote programmed cell death. In TBI patients, apoptotic‐related factors such as Bcl‐2, caspase‐1 and caspase‐3 were increased in the brain tissues, and the activities of Bcl‐2, cytochrome c and caspase‐3 were identified in the cerebrospinal fluid.^4^, ^5^, ^6^, ^7^, ^8^ However, there might be still several apoptotic‐related factors that are involved in the apoptosis after TBI.

Hence, in this study, antibody technology because of its advantages of being amenable to high‐throughput screening and rapid parallel detection of multiple proteins have been utilized to provide a clearer insight into the apoptotic mechanism after TBI, and further in vitro experiments were designed to prove it.

## 2 MATERIALS AND METHODS

### 2.1 The establishment of TBI rats

Thirty Sprague‐Dawley rats (weighing 300‐350 g, and purchased from the Chinese People's Liberation Army Medical Center Experimental Animal Center [SCXK‐(Army)‐2007‐004)] were subjected to vertical incisions over the cranium after anaesthetizing with 50% chloral hydrate. A burr hole at the junction 5 mm posterior to the coronal suture and 5 mm to the right of the sagittal suture was created to expose the dura mater. A strike was made onto the dura mater at a 3 mm depth and 5 m/s rate after randomly fixing the fifteen rats in the electron cortical contusion impactor (eCCI 6.3; Custom Design and Fabrication, Richmond, VA, USA), and this was considered as the TBI group. The other fifteen rats were included in the Sham group. Finally, all the incisions in rats were sutured. The experimental procedures were approved by the Animal Ethics Committee of the Academy of Military Medical Sciences.

### 2.2 Evaluation of Neurological Severity Scale

All rats were observed for neurological functional deficits at 6, 24, 48 and 72 hours after TBI according to the Neurological Severity Scale (NSS). This NSS evaluation includes motor function, sensory function, balance capacity and reflexes (details in Table 1). The maximum neurological score of each item is 18 points, wherein a score of 13‐18 points represents severe injury, 7‐12 points indicate moderate injury, and 1‐6 points represent mild injury.

**TABLE 1:** Neurological Severity Scale content

Signs	Description score
Motor function	When held by tail: Forelimb flexion; Hindlimb flexion; Angle of head moving basing on the vertical axis greater than 10° in 30 s Moving on horizontal surface: Normally walking; Can not straight walk; Constant circling towards paretic side; Tumbling towards paretic side
Sensory function	Orienteering test (vision and tactile); Proprioception test (deep feeling)
Balance capacity	Staying and walking parallel on the beam; Staying on the beam, with hindlimb hanging; Staying on the beam, with hindlimbs hanging or circling for more than 60 s; Falling off with attempt to stay on the beam for more than 40 s Falling off with attempt to stay on the beam for more than 20 s Falling off within 20 s with attempt to stay on the beam
Reflexes	Auricular reflex (head shaking when stimulating the ear canal); Corneal reflex (blinking when touching the cornea with cotton swab); Startle reflex (moving when hearing short and sharp voice); Epilepsy or dystonia

### 2.3 Histological test

At 72 hours after TBI, the cortices were isolated from the brain after the rats were anaesthetized and then were fixed in 4% paraformaldehyde. The fixed cortex samples were embedded in paraffin, cut into 4 μm sections and then stained with haematoxylin and eosin (HE). Histology was observed in a blinded manner by using a BX53 light microscope (Olympus Corporation, Japan) at 400× magnification.

### 2.4 Measurement of caspase‐3 activity

The caspase‐3 activity in the cortex tissues was determined by using a caspase‐3 activity kit (Beyotime Institute of Biotechnology, China) according to the manufacturer's protocol. Briefly, the total proteins were extracted from the cortex tissues, followed by incubation in an assay buffer containing 10 mmol/L DTT and 0.2 mmol/L caspase substrate, and DEVD‐pNA for 2 hours at 37°C. Finally, the results were measured at an absorbance of 405 nm with a microplate reader.

### 2.5 Antibody array performance

Total proteins from non‐fixed cortex samples were used for the measurement of apoptotic factors using Human Apoptosis Antibody Arrays (RayBio^®^ G‐Series Human Apoptosis Antibody Array 1; RayBiotech, Norcross, GA), which simultaneously detected 43 apoptotic factors according to the manufacturer's instructions. Briefly, 500 μg/mL protein extracts diluted by blocking buffer were added to the antibody array pools for overnight incubation. After washing, a biotin‐conjugated anti‐cytokine mix was added to combine with another site of apoptotic factors from the cortex samples to form sandwich detection. Finally, Cy3‐conjugated streptavidin was used to detect the apoptotic factor signals, which was exposed by an InnoScan 300 Microarray Scanner (Innopsys, France). The signal values were read by Mapix software and were normalized using an internal positive control that was produced by the RayBiotech analysis tool, which is specifically designed to analyse the data of Human Apoptosis Antibody Array.

### 2.6 The establishment of oxygen and glucose deprivation/reoxygenation (OGD/R) cell model

HT22 cells (American Type Culture Collection) were seeded into 6‐well plates (3 × 10^5^ cells/well) and 96‐well plates (5 × 10^3^ cells/well) and cultured with DMEM containing 10% FBS (Sigma‐Aldrich Co.) at 37°C. After 24 hours, the medium was replaced with glucose‐free and serum‐free DMEM. The cell plates were then placed in the hypoxic sealed box with a supply of 95% N_2_ and 5% CO_2_ for 20 minutes. Subsequently, the hypoxic sealed box was incubated in a humidified incubator at 37°C for 12 hours. The cells were cultured at 37°C for another 12 hours supplied with oxygen.

### 2.7 Down‐regulation of TNF‐α by RNA interference

Small interfering RNAs that specifically target TNF‐α (sense: 5′‐CUUCUACCGAUGGUUGAAA‐3′, antisense: 5′‐UUUCAACCAUCGGUAGAAG‐3′) and scrambled siRNAs (sense: 5′‐GAUCAUACGUGCGAUCAGA‐3′, antisense: 5′‐UCUGAUCGCACGUAUGAUC‐3′) were purchased from GenePharma. The OGD/R cells were transfected with siRNAs using GeneMute siRNA transfection reagent (SignaGen Laboratories) and incubated for 6 hours. DMEM with 10% FBS was added to each well and incubated for 24 hours again.

### 2.8 Western blot analysis

OGD/R cells were collected from 6‐well plate, and the protein was extracted using RIPA lysis buffer. 50 μg of total protein underwent 10% SDS‐PAGE electrophoresis and then was transferred onto the polyvinylidene difluoride membrane. The membrane was incubated with anti‐mouse TNF‐α and anti‐mouse p53 monoclonal antibodies (Abcam) overnight at 4°C and then cultivated with anti‐IgG‐HRP at room temperature for 2 hours. After incubating the Immobilon Western Chemiluminescent HRP Substrate (EMD Millipore), the signals were exposed using a chemiluminescence imager (Bio‐Rad).

### 2.9 Cell viability assay

Cell Counting Kit‐8 solution (Beyotime Company, China) was added into 96‐well plates, reaching to 10 μL/well. The 96‐well plates were then cultured at 37°C containing 5% CO_2_ for 1 hour. The plates were read at 450 nm using a microplate reader (Bio‐Rad).

### 2.10 Flow cytometry assay

The cells were collected from the 6‐well plates and then were incubated with Annexin V‐FITC at a final concentration of 100 ng/mL in the dark for 10 minutes. After washing with PBS, propidium iodide (PI) was used to stain the cells. Flow cytometric analysis was performed for cell apoptosis using a Becton Dickinson FACStar plus flow cytometer according to the instructions of the manual from Becton Dickinson. Data analysis was performed with standard CellQuest software (Becton Dickinson).

### 2.11 Statistical analysis

Statistical analyses were performed by Student's *t* test using SPSS v.17.0 (SPSS Inc, Chicago, IL), and all data are presented as means ± SD. The two‐sided *P* values of <0.05 were considered to be significantly different. In addition, fold change (FC) values between the two groups were calculated to indicate the relative expression levels of apoptotic factors. For antibody array detection, the average signal value of each protein in each group with >150 was considered positive expression.

## 3 RESULTS

### 3.1 The results of NSS evaluation

**FIGURE 1:**
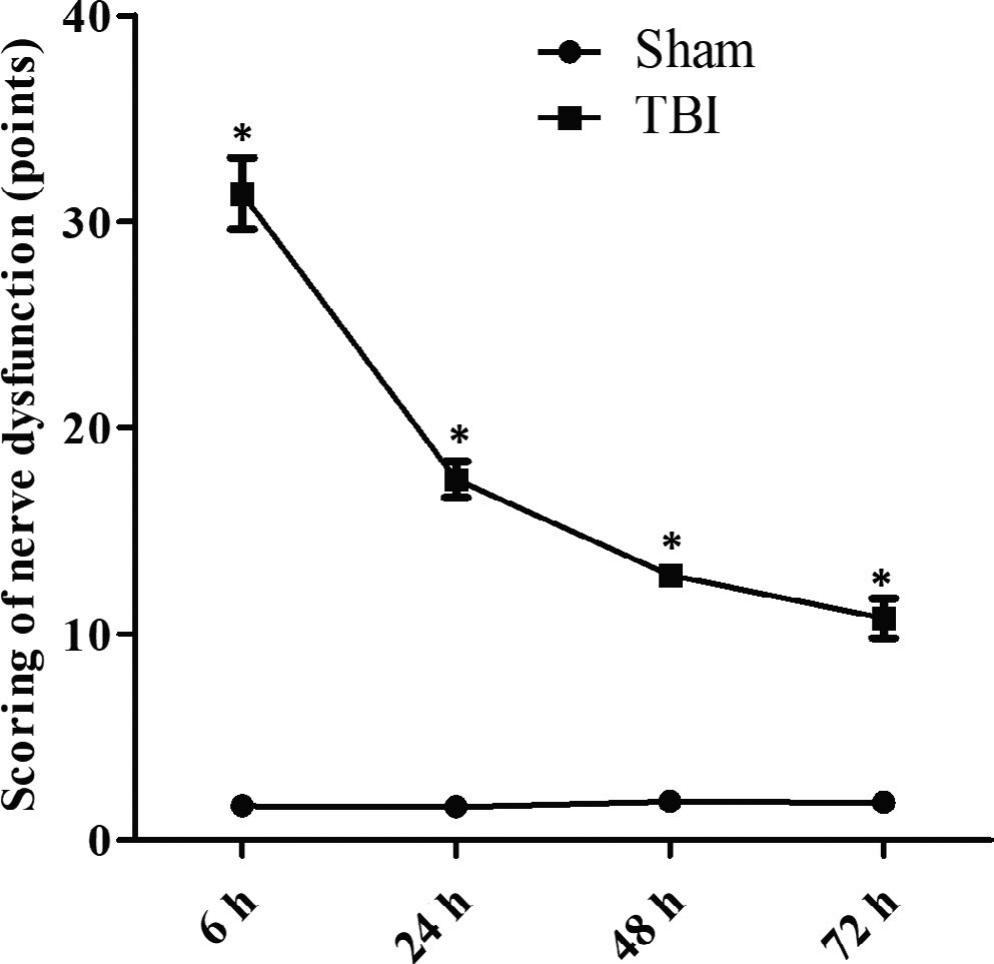
The Neurological Severity Scale (NSS) score of nerve dysfunction in rats. The NSS scores were evaluated at 6, 24, 48 and 72 h after traumatic brain injury (TBI). Data are presented as means ± SD. *P < 0.05 vs TBI group for the same time point. n = 15 in each group. The experiment was performed once

After TBI, the symptoms including contralateral forelimb flexion, tilting towards the contralateral side and other neurobehavioural changes were observed in the TBI group. The NSS scores of mice in the TBI group were significantly higher than that of Sham group at different time points (Figure 1).

### 3.2 Histological observation

**FIGURE 2:**
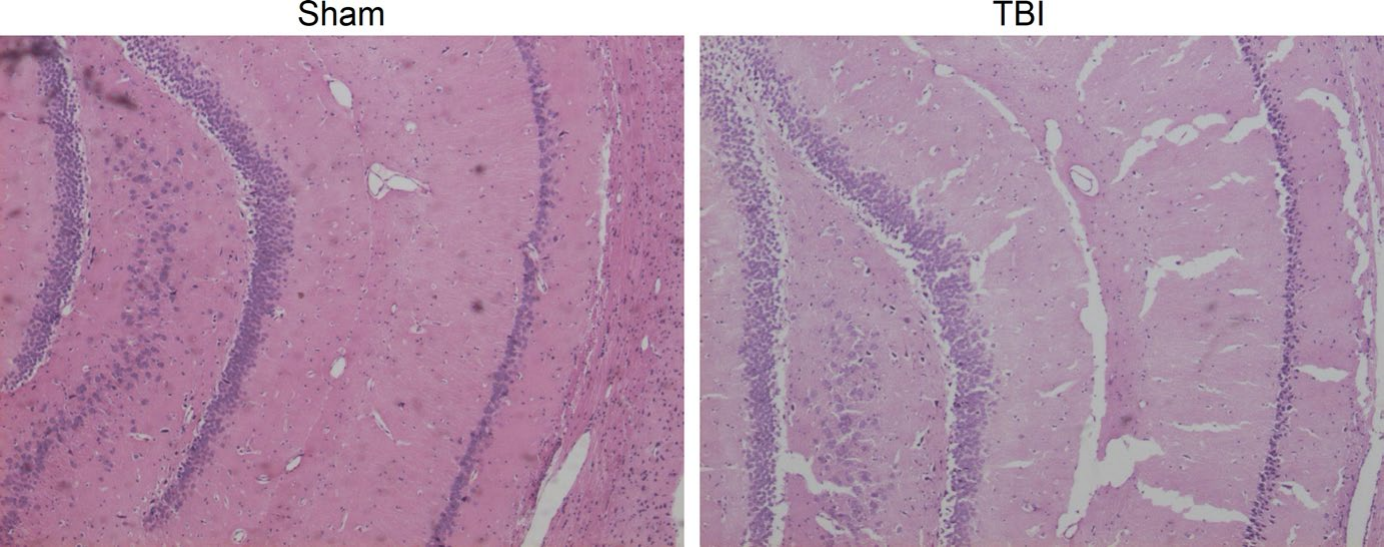
Haematoxylin and eosin (HE) staining results. The cell morphology of brain cortices at 72 h after traumatic brain injury (TBI) was detected, and HE staining showed extensive edema, neuronal necrosis, karyolysis and vacuolar changes, and widened intercellular gaps in brain cortices. The experiment was performed once

At 72 hours after TBI, HE staining of brain cortices was performed. As shown in Figure 2, the morphological structure of nerve cells was normal in Sham group. While in TBI group, patchy haemorrhages, edema, varied cell morphologies, vacuole‐like changes, neuronal degeneration and apoptosis were obviously observed in brain cortices.

### 3.3 TBI decreases caspase‐3 activity

**FIGURE 3:**
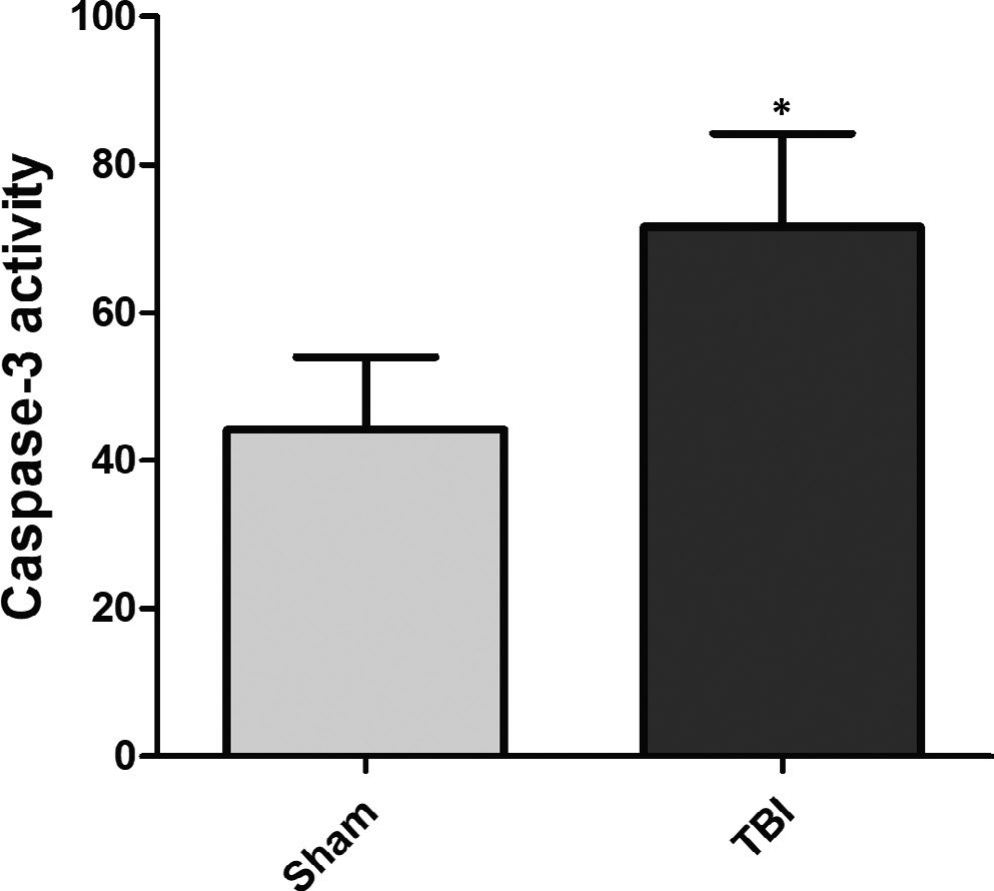
Capase‐3 activity. The caspase‐3 activity was increased in traumatic brain injury (TBI) group compared with Sham group. *P < 0.05 vs Sham group. n = 15 in each group. The experiment was performed once

To evaluate whether TBI induces apoptosis in brain tissues, the caspase‐3 activity was measured. As shown in Figure 3, the caspase‐3 activity in TBI group was significantly higher than that in Sham group.

### 3.4 Differential apoptotic factors

**FIGURE 4:**
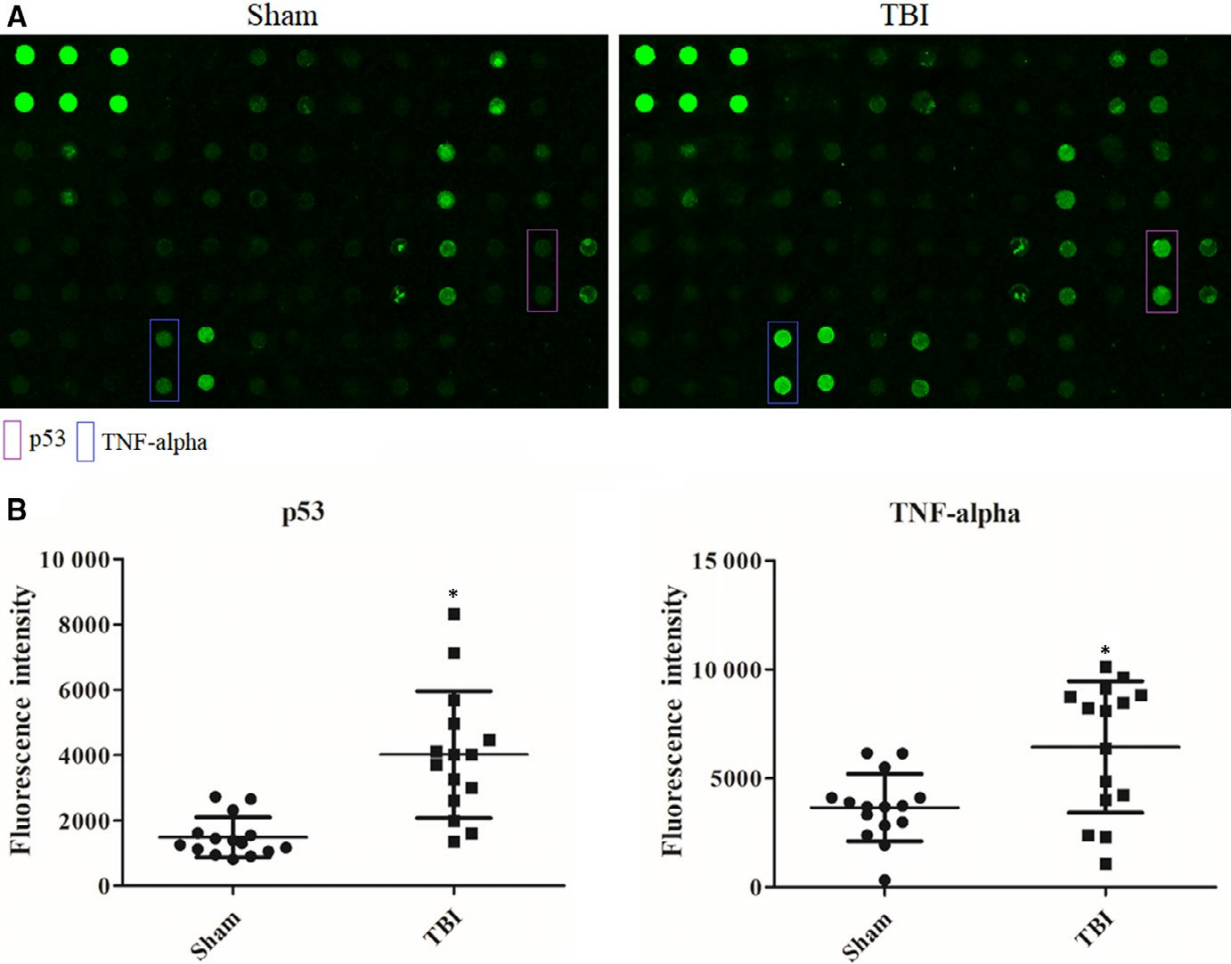
A, Antibody array profiles. The locations of the four differential proteins in antibody array profiles were labelled with coloured rectangle. The stronger signals in antibody array profiles mean higher expression levels. B, Scatter diagram. The fluorescent signals of these four proteins were transformed into the signal values, and then, the values are shown by scatter diagram. *P < 0.05 vs Sham group. Data are presented as means ± SD. n = 15 in each group. The experiment was performed once

To reveal the apoptotic mechanism after TBI, 43 apoptotic‐related proteins were detected. As shown in Figure 4A, the fluorescent signals of TNF‐α and p53 in the array profile of TBI group were brighter than those in the Sham group. Likewise, the fluorescent values of these proteins in TBI group were significantly higher than those in Sham group after transformation with fluorescent signals (Figure 4B). As known, the signal intensities of proteins in the antibody array showed positive association with their expression levels. Therefore, the levels of TNF‐α and p53 were increased after TBI.

### 3.5 TNF‐α expression after RNA interference

**FIGURE 5:**
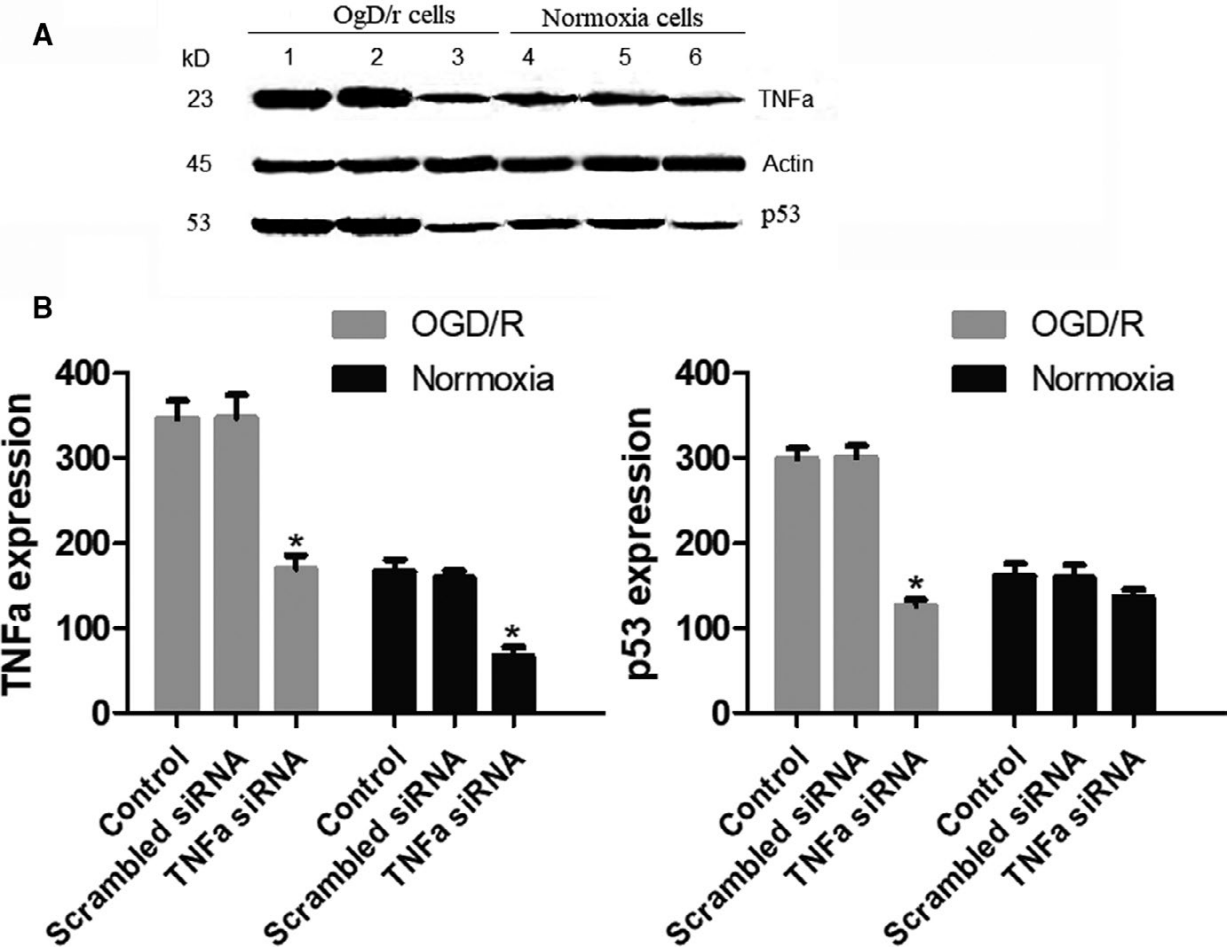
Western blot results. A, The expression profile of TNF‐α and p53 in cells by various treatments. 1. Control OGD/R cells. 2. Scrambled siRNA in OGD/R cells. 3. TNF‐α siRNA in OGD/R cells. 4. Control normoxia cells. 5. Scrambled siRNA in normoxia cells. 6. TNF‐α siRNA in normoxia cells. B, The relative levels of TNF‐α and p53 which are represented by grey values. The experiment was repeated three times

After RNA interference, the TNF‐α expression of HT22 cells was detected by Western blotting. As shown in Figure 5, the TNF‐α expression was obviously down‐regulated in TNF‐α siRNA group of normoxia cells and OGD/R cells when compared to that in scrambled siRNA and control groups. This suggested that TNF‐α siRNA treatment could inhibit the expression of TNF‐α in the cells. Furthermore, TNF‐α siRNA could significantly down‐regulate the expression of p53 in OGGD/R cells.

### 3.6 Down‐regulation of TNF‐α promoted cell viability and inhibited apoptosis in OGD/R cells

**FIGURE 6:**
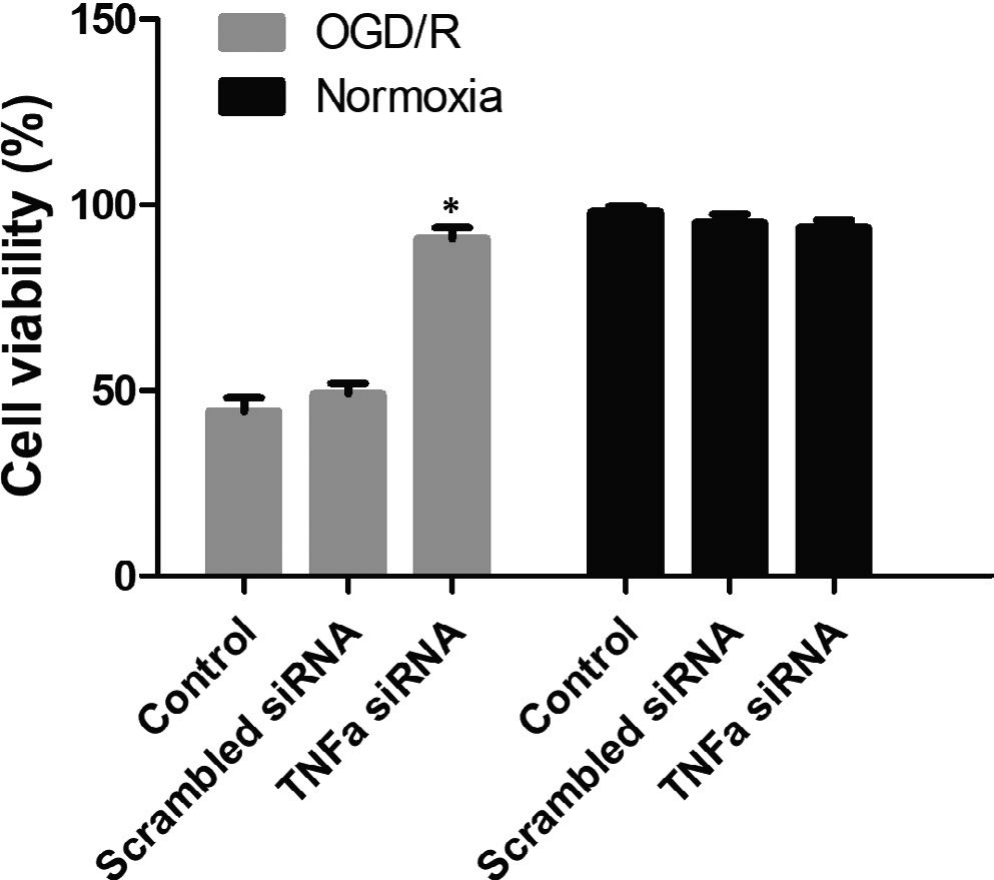
The cell viability. The cell viability of cells by various treatments was measured. TNF‐α siRNA could increase the cell viability of OGD/R cells. *P < 0.05 vs control OGD/R cells group. The experiment was repeated three times

**FIGURE 7:**
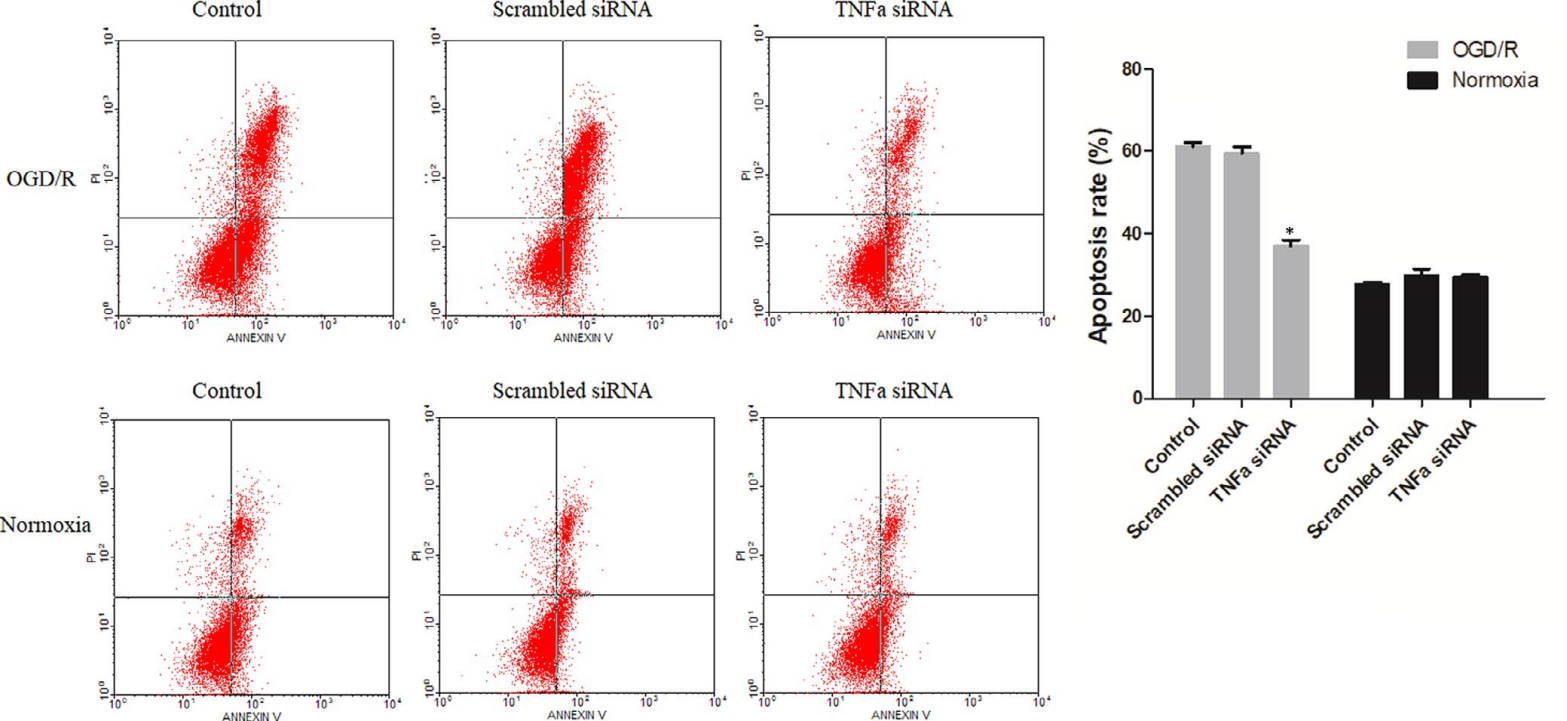
The cell apoptosis. The cell viability of cells by various treatments was measured. TNF‐α siRNA could decrease the apoptosis of OGD/R cells. *P < 0.05 vs control OGD/R cells group. The experiment was repeated three times

To study the effect of TNF‐α on OGD/R cells, cell viability and apoptosis were measured. The results revealed that cell viability was significantly reduced in OGD/R cells when compared to normoxia cells, while TNF‐α siRNA treatment notably promoted cell viability of OGD/R cells (Figure 6). In contrast, the apoptotic rate was significantly increased when the cells were under OGD/R conditions, but the apoptotic rate was evidently decreased after TNF‐α siRNA treatment in OGD/R cells (Figure 7).

## 4 DISCUSSION

Traumatic brain injury damage is induced directly by physical damage and involves complicated pathophysiological changes including primary and secondary injuries. The primary injury occurs at the moment of impact, while the secondary damage includes calcium overload, oxidative stress, inflammation and apoptosis.^9^, ^10^ Apoptosis leads to more severe neuronal death, contributing to the overall pathology of TBI, and the percentage of apoptotic neurons has been used as an index for evaluating TBI severity.^11^, ^12^

Previous researches have reported that Bcl‐2, caspase‐1, caspase‐3 and cytochrome c as important apoptotic regulators for TBI.^4^, ^5^, ^6^, ^7^, ^8^ It has been shown that apoptosis is inhibited by caspase‐3 inhibitor, in which a 30% reduction in the lesion volume was observed at 3 weeks after TBI.^13^ However, there might be more apoptotic‐related factors that are involved in the apoptosis after TBI. In the present study, the rats were impacted to establish a TBI model that was proved by increasing NSS scores and destroyed cortex tissues. Furthermore, a TBI‐induced apoptosis in the brain tissues was shown by increased neuronal apoptosis in cell morphology and increased caspase‐3 activity. To further reveal a clearer molecular mechanism of apoptosis after TBI, an apoptosis antibody array was performed to measure 43 apoptotic‐related factors. The results of this showed that the levels of TNF‐α and p53 were increased after TBI.

**FIGURE 8:**
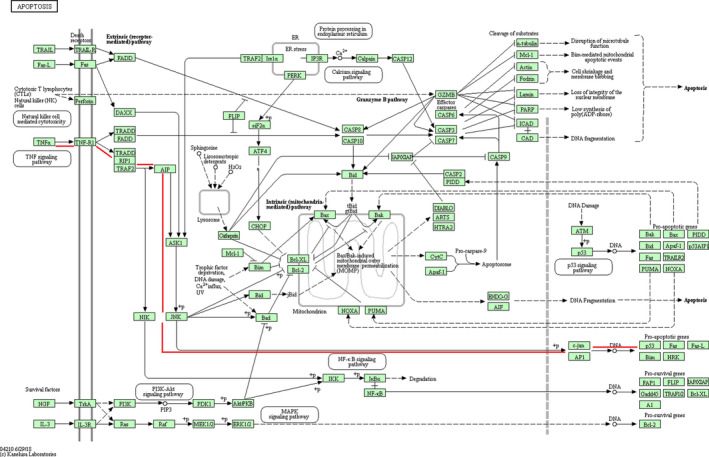
Apoptotic signalling pathway. TNF‐α and p53 involve into the same path in the apoptotic signalling pathway, which is labelled with red lines

TNF‐α normally induces cell proliferation and inflammation and induces cells into two distinct types of programmed cell deaths: apoptosis and necroptosis.^14^ However, in the present study, TNF‐α was initially found to be increased after TBI, providing evidence that TNF‐α was involved in TBI‐induced neuronal apoptosis. The p53 protein, which is a master regulator of cell apoptosis, regulates the repair of cellular DNA, induces apoptosis and participates in the regulation of apoptotic signalling pathway.^15^ What's more, p53 plays a role in the regulation of neurite outgrowth and axonal regeneration, and it is dramatically up‐regulated by oxidative stress in neurons^16^ and in experimental TBI,^17^ which was consistent with our study results. Additionally, blocking p53 protects against the neuronal loss induced by TBI.^18^ These findings suggest that p53‐induced apoptosis plays a crucial role in the pathogenesis of TBI. TNF‐α and p53 are observed in the same path of the apoptotic signalling pathway. As shown in Figure 8, in this path of apoptotic signalling pathway, TNF‐α acts as an upstream molecule, which activates the apoptotic signalling pathway by activating the downstream molecule p53 to promote cell apoptosis. Therefore, we speculated that apoptosis after TBI might be induced by the activation of this path of apoptotic signalling pathway, which was labelled with red lines in Figure 8.

To confirm this hypothesis, OGD/R cell model was established and TNF‐α down‐regulation was performed. The results showed that the cell viability of OGD/R cells was decreased, the apoptosis was promoted, and the expression of TNF‐α was up‐regulated. However, the down‐regulation of TNF‐α in OGD/R cells increased cell viability and decreased apoptosis. Furthermore, the down‐regulation of TNF‐α also decreased the expression of p53. These results suggested that oxygen and glucose deprivation/reoxygenation decreased the cell viability and increased the apoptotic rate by TNF‐α overexpression to up‐regulate the expression of p53. Previous studies have shown TNF‐α induced apoptosis and accumulation of p53 in various cell types, which suggested the potential involvement of p53 in TNF‐α–induced cell apoptosis.^19^, ^20^, ^21^, ^22^, ^23^ Consistently, the present study demonstrated that the down‐regulation of TNF‐α increased the cell viability of OGD/R cells and decreased the cell apoptosis, simultaneously reduced the expression of p53. This also suggested the possibility of involvement of p53 in TNF‐α–induced cell apoptosis.

In summary, TNF‐α and p53 were increased in the brain cortices after TBI. TNF‐α acts as an upstream molecule, and p53 acts as a downstream molecule in the same apoptotic signalling pathway, suggesting that TNF‐α activates apoptotic signalling pathway to induce apoptotic p53. This might be the apoptotic mechanism involved after TBI. Further in vitro experiments demonstrated that down‐regulation of TNF‐α in OGD/R cells could increase cell viability and decrease apoptosis and p53 expression, suggesting the involvement of molecular mechanism of neurological injury in TNF‐α–induced apoptotic signalling pathway by activating p53 (Figure 8). This needs further validation by inhibiting intermediate molecules such as the phosphorylation of JNK or c‐jun, the expression of TNF‐R1, and further research will be conducted for detailed mechanism of p53 inducing apoptosis in TBI.

## CONFLICT OF INTERESTS

The authors declare that no conflicts of interest exist.

## AUTHOR CONTRIBUTIONS

Xuefei Shao and Xiping Yang conceived and designed the experiments. Xuefei Shao, Xiping Yang, Jun Shen, Sansong Chen, Xiaochun Jiang and Qifu Wang performed the experiments. Xuefei Shao and Di Qiang analysed the data. Xuefei Shao wrote the paper. Di Qiang reviewed the paper. All authors read and approved the final manuscript.

## Data Availability

The data used to support the findings of this study are included within the article.
